# Insights into the Metabolite Profiles of Two *Camellia* (Theaceae) Species in Yunnan Province through Metabolomic and Transcriptomic Analysis

**DOI:** 10.3390/biom14091106

**Published:** 2024-09-03

**Authors:** Miao Niu, Ranyang Li, Xiongyu Li, Hongyan Yang, Jianliang Ding, Xianxiu Zhou, Yuqi He, Yawen Xu, Qian Qu, Zhiwei Liu, Jiahua Li

**Affiliations:** 1 College of Tea Science, Yunnan Agricultural University, Kunming 650201, China; 13577036525@163.com (M.N.); lxy19912992159@163.com (X.L.); 13529725893@163.com (H.Y.); 13937016027@163.com (J.D.); 2023210231@stu.ynau.edu.cn (X.Z.); 13578128508@139.com (Y.H.); 13658334948@163.com (Q.Q.); 2018016@ynau.edu.cn (Z.L.); 2 College of Horticulture, Hunan Agricultural University, Changsha 410128, China; liranyang@stu.hunan.edu.cn; 3 College of Pu-Erh Tea, West Yunnan University of Applied Sciences, Puer 665000, China; xuyawen@pecxy.edu.cn

**Keywords:** tea species evaluation, *Camellia sinensis*, *Camellia taliensis*, metabolome, transcriptome

## Abstract

Tea (*Camellia sinensis*) falls into the family Theaceae, is a valuable commercial crop, and tea products made from its buds and young leaves are favored by consumers all over the world. The more common *Thea* plant is *Camellia sinensis* (*C. sinensis*), but its most important relative, *Camellia taliensis* (*C. taliensis*), is also utilized by locals in the area of cultivation to manufacture tea. In this investigation, *C. taliensis* (DL) and *C. sinensis* (QJZ) were characterized in terms of their agronomic traits, physicochemical indices, metabolomics, and transcriptomics. The leaf area of DL is larger than that of QJZ; the color of DL’s buds and leaves is yellowish-green, while that of QJZ’s is green. DL’s buds and leaves are more densely velvety than those of QJZ. The HPLC results indicated that the physicochemical contents varied considerably between the two samples, with DL having greater concentrations of EGCG and GABA than QJZ, while QJZ had remarkably higher concentrations of C, CA, and EGC than DL. A total of 2269 metabolites and 362,190,414 genes were positively identified, with the number of DAMs and DEGs being 1001 and 34,026, respectively. The flavonoids, phenolic acids, and alkaloid metabolites were dramatically different between the two tea group plants. Bioinformatics profiling revealed that the DAMs and DEGs of the two tea group plants interacted with each other and were involved in metabolic pathways, including “biosynthesis of secondary metabolites”, “biosynthesis of amino acids”, “biosynthesis of cofactors”, “phenylpropanoid biosynthesis”, and “flavonoid biosynthesis”. Overall, these results provide statistical support for germplasm conservation and production for both *C. taliensis* and *C. sinensis*.

## 1. Introduction

Tea (*Camellia sinensis*), belonging to the family Theaceae in the section *Thea*, is a type of perennial woody evergreen plant, widespread in tropical and subtropical regions of the world, particularly in Asia [1,2,3]. The tea plant is thought to have originated in Yunnan Province in southwest China, according to research and evidence [4,5,6]. More than 50 countries and regions around the world cultivate it as a valuable economic crop [7]. Tea made from the tender buds and young leaves of the tea plant through specific processing procedures is a purely natural, green, healthy beverage. Its rich aroma, warm taste, wonderful flavor, and variety of health benefits are well received by consumers around the world [8,9,10]. Tea contains many nutrients and is a rich source of essential amino acids, antioxidants, phenols, sugars, vitamins, and minerals that the human body demands [11].

The genus *Camellia* is a large family that consists of more than 110 taxonomic groups, of which *C. sinensis* is the most commonly grown tea plant and typically used commercially as a source of beverage tea [12]. *C. sinensis* produces more mutations with varying leaf sizes, and cultivated plants usually have smaller leaves than wild native species. There are three variants of *C. sinensis*: *C. sinensis* var. *assamica* (Masters) Kitamura, *C. sinensis* var. *publimba* Chang, var. nov, and *C. sinensis* var. *waldensae* (S.Y. Hu) Chang [13]. Several species of the congeneric genus of *C*. section *Thea* are also used to make tea locally [14]. *Camellia taliensis* (W.W. Sm.) Melch, named “Dali Cha” in Chinese, known as “wild tea”, is one of the most prominent wild relatives of *C. sinensis* and is an irreplaceable germplasm resource [15]. Because of its abundance of inclusions, its leaves are widely used by locals to make tea [16]. However, very scarce research has been performed on *C. taliensis*; thus, the biosynthesis pathways and related regulatory gene mechanisms of the bioactive components of *C. sinensis* and *C. taliensis* have not yet been clarified.

Metabolomics is a novel discipline following genomics and proteomics, which can directly reflect the biological processes and mechanisms during a specific physiological period [17]. From 2000 to the present time, metabolomics has attracted increasing attention and has been widely used in the tea industry in relation to the cultivation, processing, and classification of varieties, grades, and origins [18,19,20,21]. Identifying transcripts with functional information in plant genomes can be achieved through high-throughput transcriptome analysis [22]; this method has been extensively applied in relevant studies of tea germplasm resources [23]. Moreover, the accelerating development of metabolomics and transcriptomics association technology has boosted gene expression profiling involving different metabolite pathways to excavate key functional genes [16].

Yunnan, as the origin of tea, provides unique and superior climatic and ecological conditions for the formation of rich tea germplasm resources. *C. taliensis* and *C. sinensis* have significant differences in phenotypic traits, while each has its own characteristics in biochemical composition, suitability and resistance. However, the current research on the *C. taliensis* and *C. sinensis* mainly focuses on the conventional functional components, and the lack of quality formation mechanism, characteristics and other related basic data, the absence of effective linkage between quality and histological data, and the limitations of the understanding of the specific functional components constrain the further excavation of local tea resources. In this study, we first observed and recorded the phenotypic traits of DL and QJZ, measured the routine physicochemical indices, and combined metabolomic and transcriptomic analysis to explore the molecular processes of metabolite accretion and differences in DL and QJZ. The outcomes of the investigation are intended to provide partial data support for the further exploitation and conservation of relevant germplasm resources, the expansion of production, and the enhancement of the commercial value of tea plants.

## 2. Materials and Methods

### 2.1. Plant Materials

The two tea species (*C. taliensis* and *C. sinensis*) used in this study were obtained from the tea garden of Pu’er Lancang Ancient Tea Co., Ltd., Puer, China (22°47′ N, 100°54′ E). The tea shoots (one bud with two leaves) of each tea plant were plucked at 10 AM on the 4th of May. In order to analyze the metabolomes and transcriptomes of all shoots, they were refrigerated in liquid nitrogen instantly and preserved at −80 °C.

### 2.2. Observation and Determination of Morphological Characteristics

Based on the Descriptors and Data Standards for Tea (*Camellia* spp.) [24], and the Industry Standard of China NY/T 1312-2007 [25], 13 phenotypic traits were selected for the statistics of DL and QJZ, including plant shape, growth habit, leaf size, leaf shape, bud color, bud pubescence, bud brightness, tenderness, leaf texture, leaf upper surface, leaf margin, density of leaf serration, and sharpness of leaf serration.

### 2.3. Determination of Tea Physicochemical Indices

National Standard of China GB/T 8303-2013 was followed when processing tea samples (drying, grinding, screening) [26]. The aqueous extraction, tea polyphenols, free amino acids, and catechins were measured according to previous reports. Three replications were performed for each experiment. Refer to the subsections below for further details.

#### 2.3.1. Determination of Aqueous Extraction

Based on previous research [27], in a 500 mL conical flask, 3 g (to the accuracy of 0.001 g) of the tea powder was transferred and 300 mL of boiling water was added. Following this, the conical flask was transferred to a boiling water bath for 45 min (shaking every 10 min). A vacuum filter was used to filter the hot mixture after extraction. The tea residue was rinsed several times with about 150 mL of boiling water and then the tea residue and a known mass of filter paper were transferred into an aluminum box and placed together in a constant temperature oven. The oven temperature was set at 120 °C. The aluminum box was opened and tilted to one side. After 1 h, the lid was closed, removed and cooled for 1 h, then placed the box back in the oven for 1 h. The aqueous extract content was computed using Formula (1).

(1) $$\mathrm{Aqueous}\;\mathrm{extract}\;\mathrm{content}=\left(1-\frac{m_{1}}{m_{0}\times \omega }\right)\times 100\%$$

*m*_0_—the weight of the tea powder, g;
*m*_1_—the weight of the dried tea residue, g;
*ω*—the dry matter content of the tea sample (mass fraction), %.

#### 2.3.2. Determination of Total Tea Polyphenols

The Folin–Ciocalteu method was used to measure the total tea polyphenol content [28]. For the extraction, 0.2 g of tea powder was placed in an extraction tube with 5 mL of 70% methanol, which was extracted for 10 min at 70 °C with shaking every 5 min. The methanolic extract was analyzed for tea polyphenols using a Metash (Shanghai, China) X-5 spectrophotometer at 765 nm, and the total phenolic content was calculated from the gallic acid standard curve. The results were expressed as gallic acid milligram equivalent per 100 g dry weight (GAE mg/100 g). The total tea polyphenol content was computed using Formula (2).

(2) $$Tea\;polyphenols=\frac{A\times V\times d}{{SLOPE}_{std}\times m\times 10^{6}\times \omega }\times 100$$

*A*—absorbance of test solution;
*V*—volume of sample extract, mL;
*d*—dilution factor;
SLOPE_std_—slope of the standard curve for gallic acid;
*ω*—the dry matter content of the tea sample (mass fraction), %
*m*—the weight of the tea sample, g.

#### 2.3.3. Determination of Total Free Amino Acids

The content of total free amino acids was characterized using the National Standard of China GB/T 8304-2013 [29]. In simple terms, the prepared tea infusion (1 mL), phosphate buffer (1/15MNa_2_HPO_4_, 1/15MKH_2_PO_4_, pH 8.0) (0.5 mL), and reaction solution [2% ninhydrin (C_9_H_4_O_3_·H_2_O)] (0.5 mL) were put into a 25 mL volumetric flask in sequence, and then boiled for 15 min. After cooling, the volume was fixed with distilled water. Absorbance values were read at 570 nm using a Metash X-5 spectrophotometer. The total free amino acid content was computed using Formula (3).

(3) $$\mathrm{Total}\;\mathrm{amino}\;a\mathrm{cids}=\frac{C/1000\times V_{1}/V_{2}}{m\times w}\times 100\%$$

*C*—the theanine weight, which could be obtained according to the OD_570_ from a standard curve made by theanine as a standard component, using the same method as mentioned above, mg;
*V*_1_—the total volume of the tea infusion, mL;
*V*_2_—the volume of the infusion taken to reaction, mL;
*m*—the dry weight of the tea sample, g;
*w*—the dry ratio of the tea sample, %.

#### 2.3.4. Quantification of Catechins and Caffeine via HPLC

Catechins and caffeine were determined by high-performance liquid chromatography (HPLC, Agilent Technologies Inc., Santa Clara, CA, USA); the adjusted program based on previous studies was as follows [30]: Weigh 0.1 g of ground tea sample, add 70% methanol (10 mL), refrigerate for 24 h, centrifuge the solution at 4500 rpm for 10 min, and filter through a 0.45 μm membrane to obtain the solution to be tested. The column used for HPLC analysis was a Poroshell 120 EC-C18 (4.6 × 5 mm, 2.7 μm, Agilent Technologies Inc., Santa Clara, CA, USA). 

The two mobile phases used in the experiments were solvent A (0.261% phosphoric acid, 5% acetonitrile) and solvent B (80% methanol). The elution gradient was 0–16 min, 92% A; 6–22 min, 50% A; 22–25.9 min, 35% A; 25.9–29 min, 0% A; 29–30 min, 0% A; the program was stopped for 30 min and then run for 6 min at 92% A.

#### 2.3.5. Quantification of Amino Acids Using HPLC

Amino acids were determined by high-performance liquid chromatography (HPLC, Agilent Technologies Inc., CA, USA); the adjusted program based on previous studies was used, as follows [31]: Weigh 1 g of ground tea sample, add 80 mL of boiling water, 80 °C water bath extraction for 1 h; after cooling, filter to 100 mL volumetric flask capacity, take 1 mL of the tea broth and add 200 μL chloroform oscillation extraction; maintain for 10 min, centrifuge at 12,000 rpm for 10 min, collect the supernatant, and filter through a 0.45 μm membrane to obtain the solution to be tested. The column used for HPLC analysis was Venusil AA (4.6 × 250 mm, 5 μm, Agilent Technologies Inc., Santa Clara, CA, USA). The two mobile phases used in the experiments were solvent A (20 μM sodium acetate, 0.018% triethylamine, 0.3% tetrahydrofuran, 0.004% ethylenediaminetetraacetic acid disodium salt dihydrate, pH 7.2) and solvent B (40% acetonitrile, 40% methanol, 0.3% acetic acid). The elution gradient was 0–2 min, 78% A; 2–12 min, 73% A; 12–35 min, 70% A; 35–39 min, 0% A; 39–39.9 min, 0% A; the program was stopped for 40 min and then run for 6 min at 92% A.

### 2.4. Widely Targeted Metabolite Identification and Quantification

#### 2.4.1. Dry Sample Extraction

Using vacuum freeze-drying technology, the biological samples were placed in a lyophilizer (Scientz-100F, Scientz, Ningbo, China) and then the samples were ground (30 Hz, 1.5 min) to powder using a grinder (MM 400, Retsch, Verder Shanghai Instruments and Equipment Co., Ltd., Shanghai, China). Next, 50 mg of sample powder was weighed and 1200 μL of −20 °C pre-cooled 70% methanolic aqueous internal standard extract was added (less than 50 mg added at the rate of 1200 μL extractant per 50 mg sample). The sample was vortexed once every 30 min for 30 s, for a total of 6 times. After centrifugation (rotation speed 12,000 rpm, 3 min), the supernatant was aspirated, and the sample was filtered through a microporous membrane (0.22 μm pore size) and stored in an injection vial for UPLC–MS/MS analysis.

#### 2.4.2. UPLC Conditions

The sample extracts were analyzed using an UPLC-ESI-MS/MS system (UPLC, ExionLC™ AD, https://sciex.com.cn/ (accessed on 8 August 2023)) and a tandem mass spectrometry system (MS/MS). The analytical conditions were as follows: UPLC column, Agilent SB-C18 (1.8 µm, 2.1 mm × 100 mm). The mobile phase consisted of solvent A: pure water with 0.1% formic acid, and solvent B: acetonitrile with 0.1% formic acid. The elution gradient was 0–9 min, 5% A and maintained for 1 min; 10–11.1 min, 95% A and maintained for 2.9 min.

#### 2.4.3. ESI-Q TRAP–MS/MS

The ESI source operation parameters were as follows: source temperature 500 °C; ion spray voltage (IS) 5500 V/−4500 V; ion source gas I, gas II, and curtain gas were set at 50, 60, and 25 psi, respectively; the collision-activated dissociation (CAD) was high. Triple-quadrupole (QQQ) scans were acquired as multiple reaction monitoring (MRM) experiments with collision gas (nitrogen) set to medium. Declustering potential (DP) and collision energy (CE) for individual MRM transitions was carried out with further DP and CE optimization. A specific set of MRM transitions was monitored for each period according to the metabolites eluted within this period.

#### 2.4.4. Data Analysis

Based on the local metabolic database, the metabolites of the samples were qualitatively and quantitatively analyzed using mass spectrometry. The MRM metabolite detection multi-peak map shows the substances that can be detected in the sample, with each color-coded mass spectrum peak representing one metabolite detected. Metabolites with VIP (variable importance) > 1, fold change ≥ 2, and fold change ≤ 0.5 were considered to have undergone significant changes.

### 2.5. RNA Sequencing (RNA-seq) Analysis and Differentially Expressed Genes (DEGs)

Three shoot samples for each SWC treatment were used for RNA-Seq. An amount of l μg RNA per sample was used as input material for the RNA sample preparations. Sequencing libraries were generated using the NEBNext UltraTM RNA Library Prep Kit for lluminaR (Orlando, FL, USA) following the manufacturer’s recommendations, and index codes were added to attribute sequences to each sample. The library preparations were sequenced on an Illumina platform and 150 bp paired-end reads were generated.

At this stage, fastp was used to filter the original data, mainly to remove reads with adapters; these paired reads are removed when the N content of any sequencing read exceeded 10% of the number of bases in the read or when the number of low-quality (Q ≤ 20) bases exceeded 50% of the number of bases in the read. All subsequent analyses were based on clean reads.

The reference genome and its annotation files were downloaded from the designated website, and HISAT was used to construct the index and compare the clean reads to the reference genome.

DESeq2 was used to analyze the differential expression between the two groups, and the *p*-value was corrected using the Benjamini–Hochberg method. The corrected *p*-value and log2foldchange were used as the threshold for significant difference expression. Functional enrichment was analyzed using Gene Ontology (GO) and the Kyoto Encyclopedia of Genes and Genomes (KEGG) database.

## 3. Results

### 3.1. Morphological Characteristics

**Figure 1:**
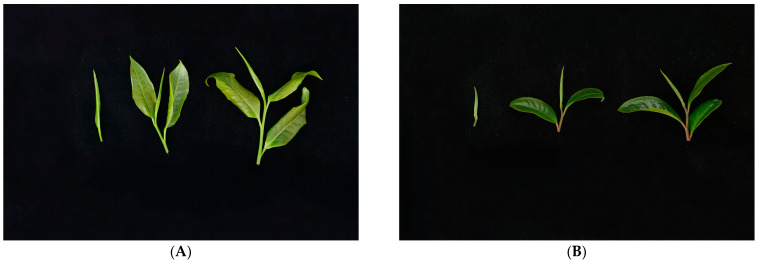
Agronomic traits of a single bud, one bud with two leaves, and one bud with three leaves from (**A**) *C. taliensis* (DL) and (**B**) *C. sinensis* (QJZ).

For the systematic analysis of the morphological characteristics of *C. taliensis* (DL) and *C. sinensis* (QJZ), agronomic traits, such as plant shape and leaf shape, were investigated. The results are as follows. The plant shape for both plants was the semi-arbor type, but DL spread horizontally and QJZ was semi-erect. The leaf size was large and medium for DL and QJZ, respectively. The leaf shape for both DL and QJZ was elliptic. The buds of DL were yellowish-green, while the buds of QJZ were green. The bud pubescence of DL was dense, while that of QJZ, on the contrary, was sparse. Both DL and QJZ had shiny buds and leaves. The tenderness of DL was stronger than that of QJZ. QJZ had a harder leaf texture compared to DL. DL exhibited a rugose leaf upper surface and wavy leaf margins, while QJZ had a slightly rugose leaf upper surface and flat leaf margins. Both plants exhibited sparse and flat leaf serration, with the difference being that the leaf serration of DL was obtuse and the sharpness of the leaf serration of QJZ was medium (Figure 1A,B).

### 3.2. Physicochemical Indices in DL and QJZ

**Figure 2:**
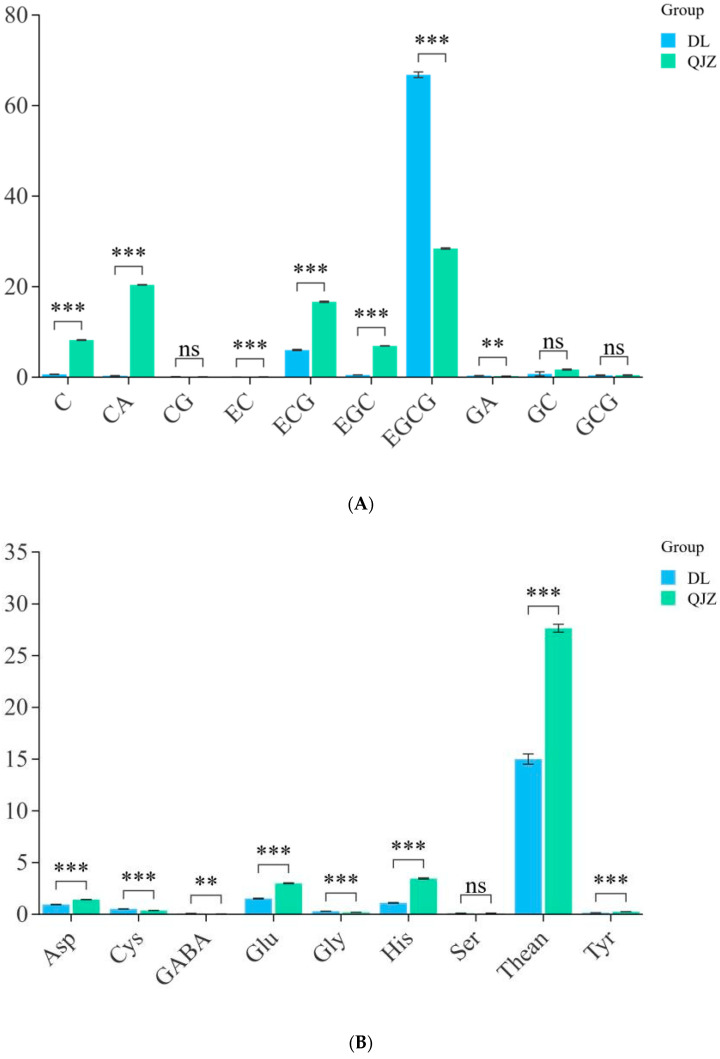
Physicochemical indices of *C. taliensis* and *C. sinensis*. (**A**) Content of catechin fraction; (**B**) Content of amino acid. ** indicates *p* > 0.001; *** indicates *p* < 0.001; “ns” indicates *p* > 0.05; C: catechin; CA: caffeine; CG: catechin gallate; EC: epicatechin; ECG: epicatechin gallate; EGC: epigallocatechin; EGCG: epigallocatechin gallate; GA: gallic acid; GC: gallocatechin; GCG: gallocatechin gallate; Asp: aspartic acid; Cys: cysteine; GABA: *γ*-aminobutyric acid; Glu: glutamic acid; Gly: glycine; His: histidine; Ser: serine; Thean: L-Theanine; Tyr: tyrosine.

To identify the major physicochemical indices of taste in DL and QJZ, we determined the aqueous extraction, tea polyphenols, free amino acids, and catechins in the two tea samples. The outcomes suggested that there were no apparent variance in terms of aqueous extraction or tea polyphenols between DL and QJZ. The quantitative analysis of catechins, caffeine, and amino acids in the tea samples were performed using the HPLC method. As shown in Figure 2, there were differences in individual components between the two tea samples. Compared with QJZ, the content of C, CA, EC, ECG, EGC, GC, Rutin, Glu, His, Phe, Thean, Thr, and Tyr were lower in DL. Notably, the content of C, CA, EGC, EGCG, and GABA showed significant differences between DL and QJZ. The content of C, CA, and EGC in QJZ were 13.03-, 64.81-, and 13.59-fold higher than those in DL, respectively. However, in DL, the EGCG and GABA contents were 2.35 times and 2.66 times higher than in QJZ (Figure 2). The discrepancy in these physicochemical indices was more dramatic in DL and QJZ. Accordingly, we initially speculated that the two species of tea have certain differences in biochemical and taste quality, which paved the way for further research to investigate the possible mechanisms of tea taste formation.

### 3.3. Metabolome Profiling

**Figure 3:**
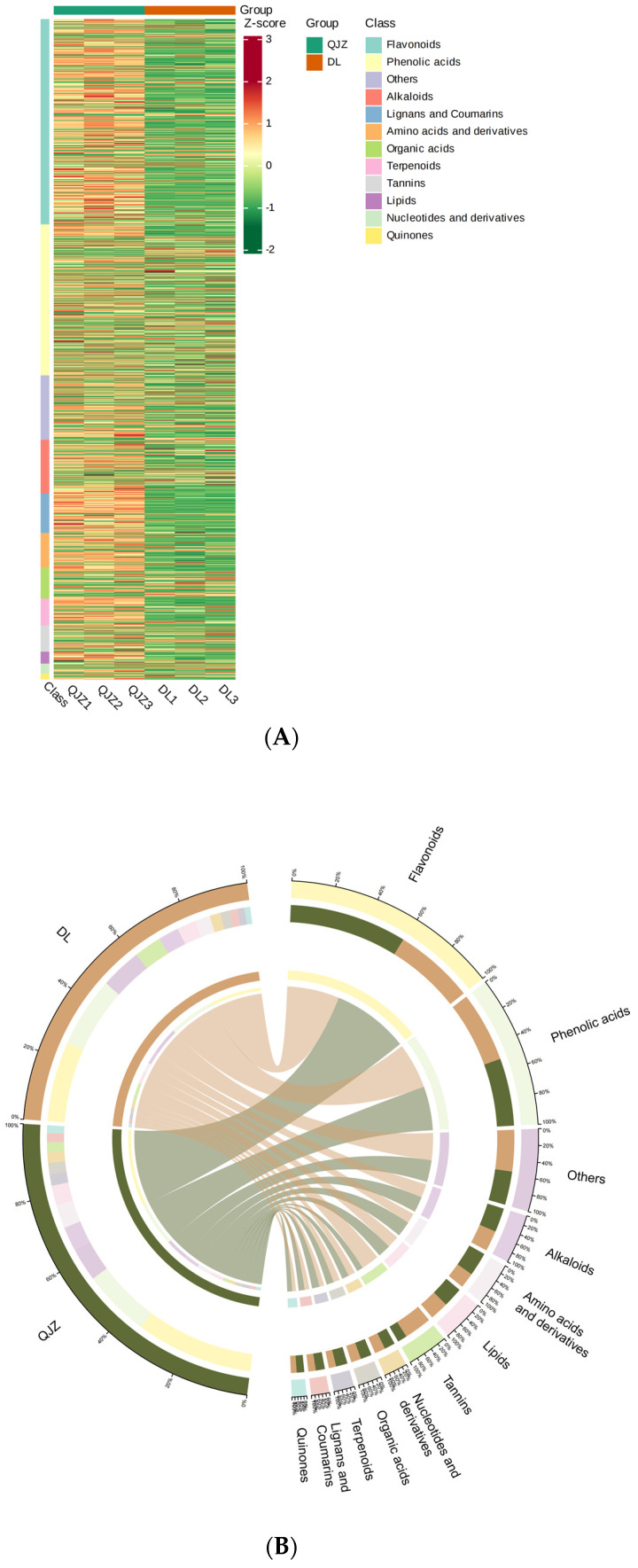
Metabolomic profiling of *C. taliensis* and *C. sinensis*. (**A**) Heat map of metabolites. Sample names are shown horizontally and metabolite information vertically. (**B**) Metabolite classification Circos chart.

To obtain the metabolite profiling within *C. taliensis* and *C. sinensis*, metabolomics was performed and 2269 individual metabolites were positively identified in these two tea samples (Figure 3A), and grouped to 12 main categories (Figure 3B): alkaloids (8.73%), amino acids and derivatives (7.89%), flavonoids (22.52%), lignans and coumarins (5.38%), lipids (6.96%), nucleotides and derivatives (3.57%), organic acids (4.89%), others (13.18%), phenolic acids (17.85%), quinones (0.84%), tannins (3.13%) and terpenoids (5.07%). The most plentiful metabolites were flavonoids, with phenolic acids, others, alkaloids, amino acids and their derivatives being the next in order.

**Figure 4:**
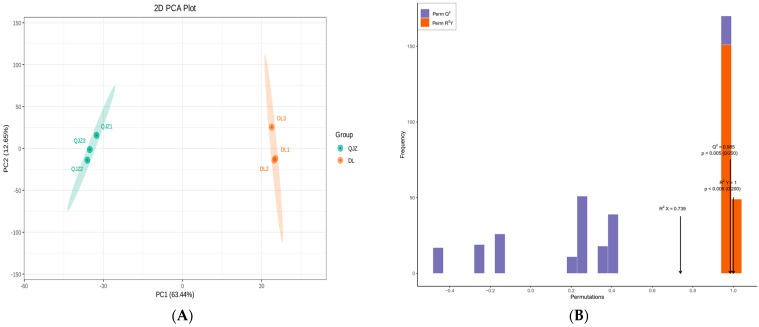
Multivariate statistical analysis of metabolites from *C. taliensis* and *C. sinensis*. (**A**) PCA score plot. (**B**) The permutation analysis of the OPLS–DA model.

The PCA (Principal Component Analysis) illustrated that PC1 and PC2 accounted for 63.44% and 12.65% of the total variance, respectively, and distinguished between the two tea samples clearly with different colors (Figure 4A). Larger value of PC1 signified a stronger incidence of genetic variation between species. The OPLS–DA (Orthogonal Partial Least Squares–Discrimination Analysis) score plot (Figure S1) also revealed significant separateness among the judgment classes of DL and QJZ. The values for *R*^2^*Y* and *Q*^2^ were 1 and 0.985, demonstrating that the metabolomic data were credible (Figure 4B).

### 3.4. Differentially Accumulated Metabolites (DAMs) Analysis of Two Tea Species

**Figure 5:**
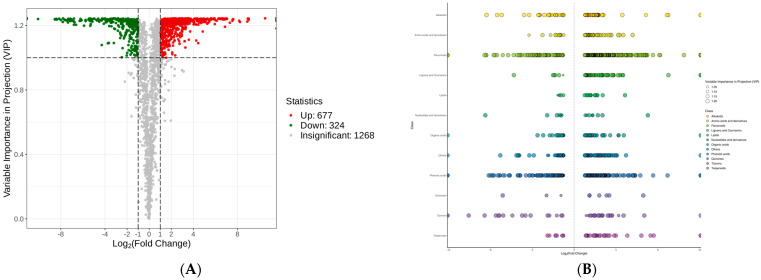
Differentially accumulated metabolite (DAM) profiles of *C. taliensis* and *C. sinensis*. (**A**) Volcano plot. (**B**) Scatter plot.

To further investigate the possible metabolic pathways involved, a volcano plot was mapped to elucidate the DAMs (Figure 5A). Under the threshold of VIP ≥ 1, fold change (FC) ≥ 2, or FC ≤ 0.5, a total of 1001 DAMs were identified, including 677 up-regulated DAMs and 324 down-regulated DAMs (Table S1). These metabolites consisted mainly of flavonoids, phenolic acids, and alkaloids (Figure 5B).

**Figure 6:**
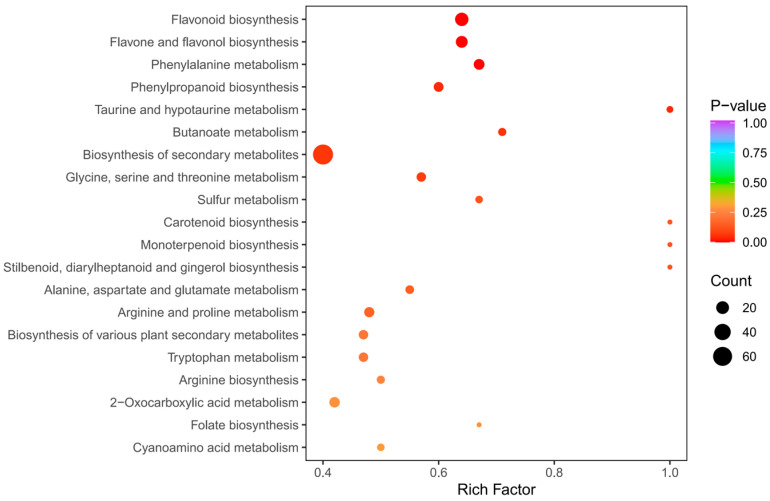
Pathway enrichment analysis of DAMs of *C. taliensis* and *C. sinensis*.

The KEGG enrichment analysis was conducted to establish the primary pathways of DAMs in *C. taliensis* and *C. sinensis*. The findings pointed that the DAMs in DL and QJZ were predominantly enriched in the “biosynthesis of secondary metabolites (ko01110)”, “flavonoid biosynthesis (ko00941)”, “flavone and flavanol biosynthesis (ko00944)”, “phenylpropanoid biosynthesis (ko00940)”, “phenylalanine metabolism (ko00360)”, “glycine, serine and threonine metabolism (ko00260)”, “taurine and hypo-taurine metabolism (ko00430)”, and “butanoate metabolism (ko00650)” pathways (Figure 6), with “biosynthesis of secondary metabolites”, and “flavonoid biosynthesis”, and “flavone and flavanol biosynthesis” being the three most prominent pathways for enrichment. The results demonstrated that these pathways and their pertinent metabolites are pivotal elements impacting the biochemical components and sensory quality of tea.

### 3.5. Comparative Transcriptome Analysis of Two Tea Species

In order to characterize the variability of performance features of *C. taliensis* and *C. sinensis* and their formation mechanisms, transcriptome sequencing and analysis were performed in this study. A total of 362,190,414 clean reads were acquired after removal of low-quality reads. The percentages of Q30 and GC were 94.4–95.09% and 45.12–45.52%, respectively, demonstrating the high standard of quality of the transcriptome sequencing data.

**Figure 7:**
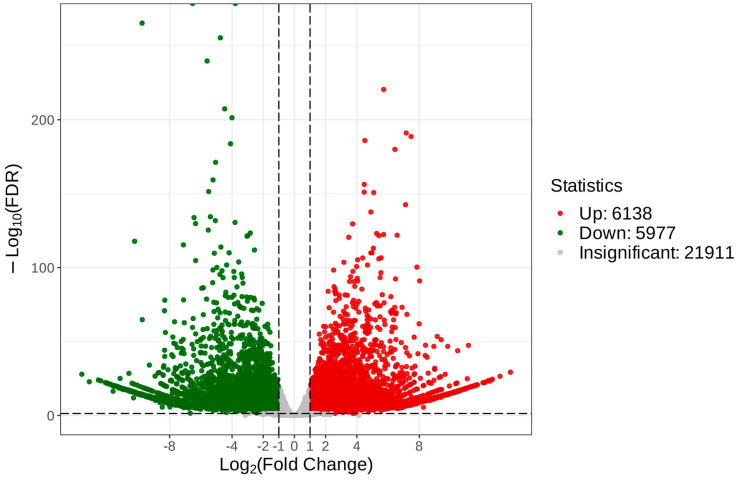
Volcano plot of DEGs of *C. taliensis* and *C. sinensis*.

To reveal the unique genes that were differentially expressed between *C. taliensis* and *C. sinensis*, we performed comparative analysis of transcriptomic data. With the thresholds of FDR < 0.05 and log2 FC ≥ 1, 34,026 DEGs were recognized (Table S2), of which 6138 and 5977 were up- and down-regulated, respectively (Figure 7).

**Figure 8:**
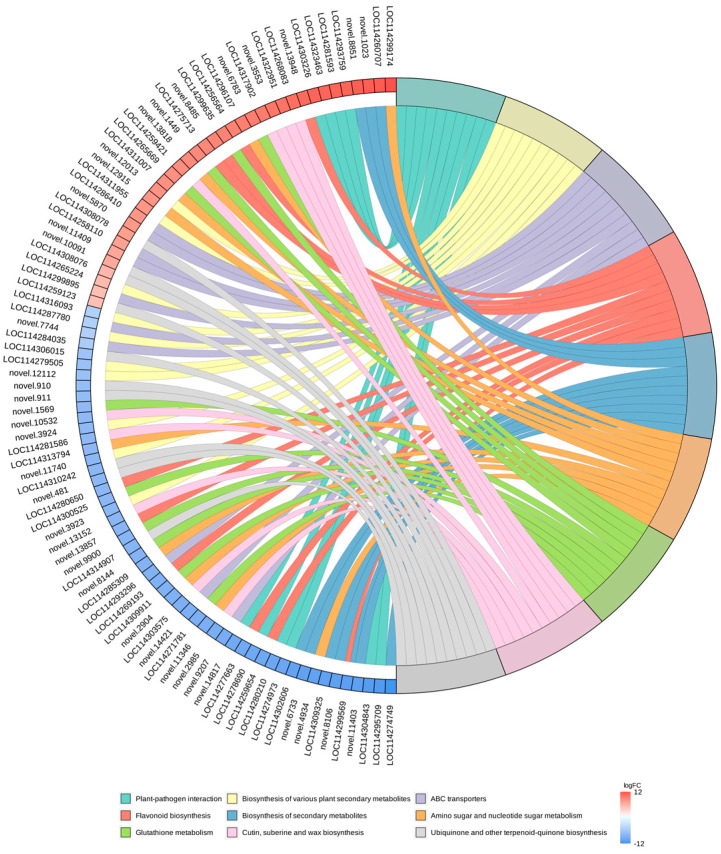
KEGG enrichment chord plot. On the left side of the graph are the 10 genes with the largest logFC in each category, on the right side of the plot are the 9 pathways with the most significant enrichment, and the central line represents the counterpart of the pathways and genes. The legend of the heatmap on the lower right side indicates the logFC values of the genes.

In this study, we selected the nine pathways with the minimum q-value and plotted the enrichment chords (Figure 8). KEGG pathway investigations pointed out that a majority of the DEGs were enriched in the “biosynthesis of secondary metabolites (ko01110)”, “biosynthesis of various plant secondary metabolites (ko00999)”, “plant–pathogen interaction (ko04626)”, “flavonoid biosynthesis (ko00941)”, “glutathione metabolism (ko00480)” and “amino sugar and nucleotide sugar metabolism (ko00520)” pathways. In parallel, we discovered that DEGs were also remarkably enriched in the “ABC transporters (ko02010)”, “protein processing in endoplasmic reticulum (ko04141)”, and “phenylpropanoid biosynthesis (ko00940)” pathways.

**Figure 9:**
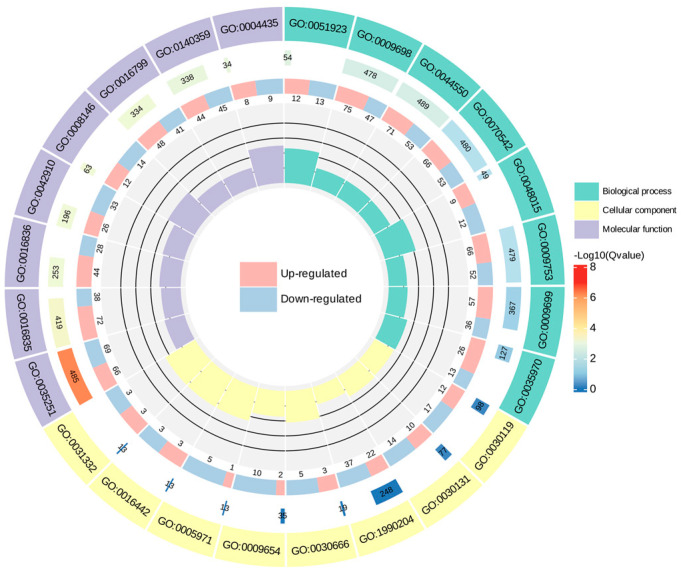
GO enrichment Circos plot of DEGs of *C. taliensis* and *C. sinensis*. In the figure, from outside to inside, the first circle denotes the GO terms; the second circle indicates the amount of background genes in that classification and q−value by bar length and color; the third circle is the up− and down−regulated gene ratio bar. Specific values are shown at the bottom; with the fourth circle representing the Rich Factor value for each classification, and all cells on the supporting line in the background correspond to 0.2.

To make a further determination of the function of the DEGs, enrichment of DEGs in the GO was analyzed. In this study, the eight GO terms with minimal q−-value in biological processes, cellular components, and molecular function were selected to map the GO enrichment Circos plot, respectively (Figure 9). The results of the GO enrichment dot plot (Figure S2) demonstrated that the nine most dramatically enriched GO terms of the DEGs were “UDP−-glucosyltransferase activity”, “carbon−-oxygen lyase activity”, “phenylpropanoid metabolic process”, “secondary metabolite biosynthetic process”, “ABC−-type transporter activity”, “antiporter activity”, “response to jasmonic acid”, “hydrolase activity, hydrolyzing N−-glycosyl compounds”, and “response to fatty acid”.

### 3.6. Conjoint Analysis of Transcriptomics and Metabolomics Data

**Figure 10:**
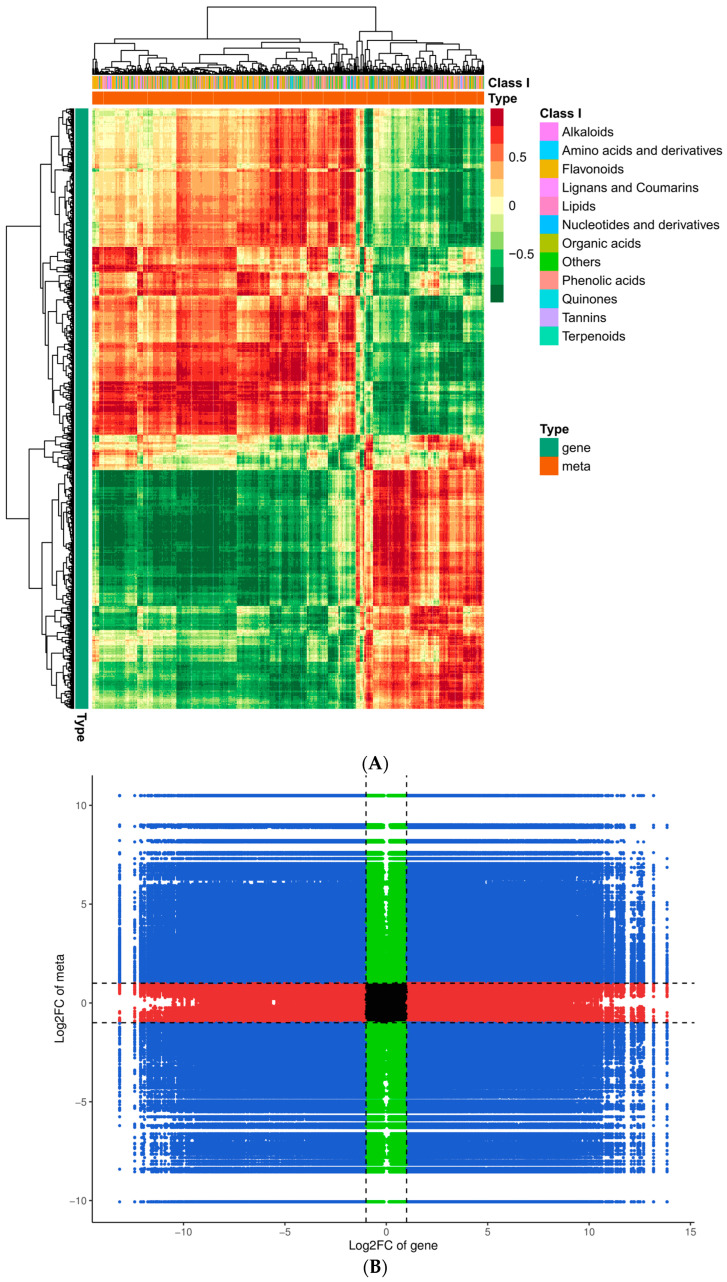

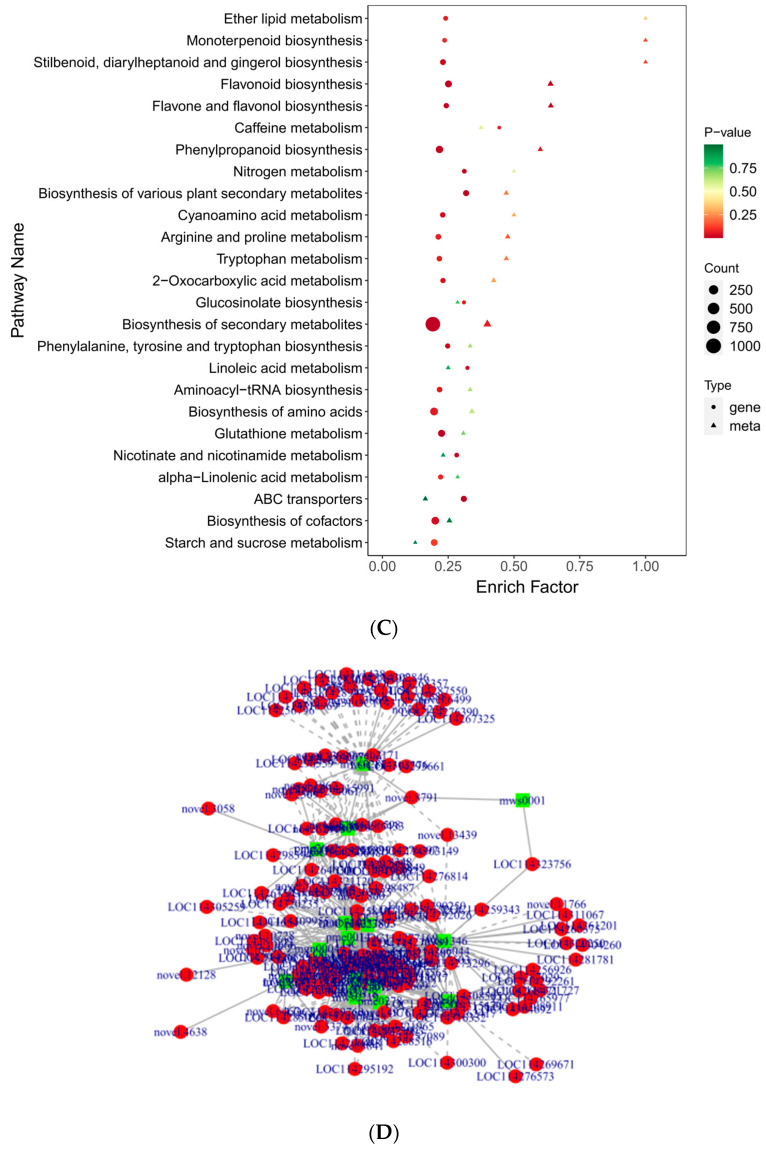
Conjoint analysis of transcriptomics and metabolomics of *C. taliensis* and *C. sinensis*. (**A**) Correlation heat map of DEGs and DAMs. (**B**) The 9−quadrant plot of DEMs and DEGs. (**C**) KEGG analysis of DEGs and DAMs that were enriched in the same pathway. (**D**) Correlation network of biosynthesis of amino acids (ko01230). Metabolites and genes are labeled with green squares and red circles, respectively. The solid and dashed lines indicate positive and negative correlations, separately.

To gain additional insight into the associations between genes and metabolites, we conducted a joint DEG and DAM analysis. Comprehensive categorization of the DEGs and DAMs was achieved by drawing a correlation heat map and categorizing the metabolites into different groups (Figure 10A). Of these, the DAMs were mainly concentrated in phenolic acids, flavonoids, alkaloids, amino acids and derivatives, and organic acids. The findings indicated significant dissimilarity between QJZ and DL. A nine-quadrant plot was drawn in accordance with the PCC > 0.8 and *p*-value < 0.05 (Figure 10B). Diverse genes/metabolites were characterized by distinct colors and quadrants as well as positive and negative regulatory interactions. Quadrant 5 indicated that neither genes nor metabolites were differentially expressed; quadrants 3 and 7 represented a consistent pattern of gene and metabolite expression, while the contrary was observed in quadrants 1 and 9. The analysis of the DAMs and DEGs in conjunction with KEGG (Figure 10C) revealed 25 co−enriched pathways in the DL and QJZ, with “biosynthesis of secondary metabolites (ko01110)”, “biosynthesis of amino acids (ko01230)”, “biosynthesis of cofactors (ko01240)”, “phenylpropanoid biosynthesis (ko00940)” and “flavonoid biosynthesis (ko00941)” being the metabolic pathways that were significantly different between the DAMs and DEGs. We also constructed correlation network diagrams by selecting DAMs and DEGs with a PCC greater than 0.80 and a *p*−value less than 0.05 in each pathway, further demonstrating the relationship between the DAMs in DL and QJZ and the DEGs enriched in the same KEGG pathway. In the biosynthesis of amino acids, 764 DAMs including 68 alkaloids, 309 amino acids and their derivatives, 281 organic acids, 55 others, and 51 phenolic acids were correlated strongly with DEGs (Figure 10D, Table S3). 

## 4. Discussion

Tea has been used in China for at least 2000 years [32]. Known as the one of the most preferred beverages around the world, tea provides health benefits, economic revenue, and employment opportunities [33,34,35]. Due to the existence of secondary metabolites, such as polyphenols, tannins, alkaloids, and amino acids, tea delivers benefits to the human body, including antioxidant, antidiabetic, anticancer, and antiallergenic properties [36,37,38,39].

Caffeine, amino acids, and polyphenols are intimately related to the quality of tea, and determines the color, taste, and aroma of tea, therefore affecting its commercial value. As one of the principal purine alkaloids in tea, caffeine is the most widely utilized psychoactive substance in the world, with pharmacological effects, such as diuresis and stimulation of the central nervous system [40]. However, precisely because caffeine has a neuro-stimulative action on the central nervous system, consumers who are sensitive to such compounds may suffer from insomnia and anxiety as a result [41]. As a result, demand has arisen for caffeine-free or decaffeinated teas. In this study, the HPLC results demonstrated that the caffeine content of DL was substantially reduced compared to QJZ, which was mutually verified with the metabolomics data. Therefore, DL can be considered as a potential decaffeinated germplasm resource for subsequent studies. Catechins are vital functional components of tea, exhibiting a wide range of biological activities, accounting for about 60–80% of total tea polyphenols [42], and are the primary ingredients in the bitter and astringent flavor of tea [43]. The concentration of catechins in QJZ was 13.03 times higher than that of DL, and it can be inferred that QJZ has a stronger, more bitter flavor. EGCG is one of the ester catechins and represents the most abundant major bioactive substance among catechins [44]. The total quantity of catechins in DL is less than in QJZ, but its EGCG content is higher, which may be caused by variations in the tea species. The two tea species also contained widely differing levels of GABA, a neurotransmitter that acts to lower blood pressure, induce relaxation, and reduce anxiety [45]. It is precisely these variations in bioactive substances that account for the shape and quality differences between the species. To develop a more in-depth understanding the mechanisms and related genes regulating these differences, metabolomics and transcriptomics profiling were made for both DL and QJZ.

The primary objective of metabolomics analysis is to examine and sift out biologically and statistically significant metabolites from samples and to illustrate mechanisms of metabolic processes and transformation in organisms [46]. In the context of the present study, a metabolomics profiling of DL and QJZ was undertaken. The experimental outcomes demonstrated that, among the 2269 metabolites detected, flavonoids, phenolic acids, others, alkaloids, and amino acids and their derivatives accounted for the majority of the metabolites, with a total proportion of more than 70% of the aggregate of metabolites, indicating that they are the principal constituents within the metabolites of tea and make a remarkable contribution to the quality of the tea leaves. The biosynthesis of secondary metabolites, flavonoid biosynthesis, and flavone and flavanol biosynthesis were the three pathways most notably enriched in the KEGG analysis.

Transcriptome broadly refers to the collection of all transcriptome products in a cell under a particular physiological condition, and the object of study refers to the combination of all of a specific cell’s RNAs that can be transcribed under a particular functional state [47]. The results suggested that most of the enriched terms belonged to molecular functions. DEGs are mainly enriched in the access pathways of “secondary metabolite biosynthesis”, “plant–pathogen interactions” and “flavonoid biosynthesis”. 

Biological processes are complex and holistic, and it is difficult to analyze the macro-developmental processes of biological systems from single-omics data [48]. Integrating multi-omics data for analysis can reduce the false positives associated with single-omics analysis and provide a panoramic window into biological activity [49]. The metabolomics and transcriptomics KEGG co-enrichment analysis showed a significant enrichment of DAMs and DEGs in the biosynthesis of secondary metabolites, biosynthesis of amino acids, biosynthesis of amino acids, and biosynthesis of cofactors, which indicated that the activities of these pathways differed dramatically between the DL and the QJZ. Through the establishment of data relationships between molecules at different levels, combined with functional analysis and metabolic pathway enrichment, we will systematically and comprehensively analyze the functions and regulatory mechanisms of biomolecules, and ultimately achieve a comprehensive understanding of the general trends and directions of biological changes, propose a model of molecular biology change mechanisms, and screen out the key metabolic pathways, or proteins, genes, and metabolites for subsequent in-depth experimental analyses and applications.

Tea quality is the consequence of the combined action of sub-metabolites, including alkaloids, tea polyphenols and amino acids [50]. Differences in tea species lead to variances in the variety and content of inclusions. Species diversity leads to differences in endogenous metabolites in the fresh leaves, which can have a dramatic effect on the flavor of the tea products, even under the same processing conditions. Precisely for this reason, the study of different tea species is the basis for a proper understanding of the suitability of the tea plant.

## 5. Conclusions

In this study, *C. taliensis* and *C. sinensis* were used as research materials to characterize and compare their phenotypic traits, biochemical components, metabolomes, and transcriptomes, revealing significant differences between them. By combining data from metabolomic and transcriptomic analysis, we found that the metabolites responsible for the differences are mainly flavonoids, phenolic acids, and alkaloids, which have an impact on the quality of tea products. These results help us to gain a deeper comprehension of the biosynthetic pathways of quality-related components in *C. taliensis* and *C. sinensis* and provide partial data support for further studies.

## Data Availability

The original contributions presented in the study are included in the article/Supplementary Materials, further inquiries can be directed to the corresponding authors.

## Supplementary Materials

The following supporting information can be downloaded at: https://www.mdpi.com/article/10.3390/biom14091106/s1, Figure S1: OPLS−DA score plot; Figure S2: GO enrichment dot plot; Table S1: The DAMs between DL and QJZ; Table S2: The DEGs between DL and QJZ; Table S3: Cornetwork file of ko01230.
