# Supplementary figures and images for: Insights into the Metabolite Profiles of Two Camellia (Theaceae) Species in Yunnan Province through Metabolomic and Transcriptomic Analysis
Source: Biomolecules. 2024 Sep 3;14(9):1106. doi: 10.3390/biom14091106 (PMC11430766; doi:10.3390/biom14091106)

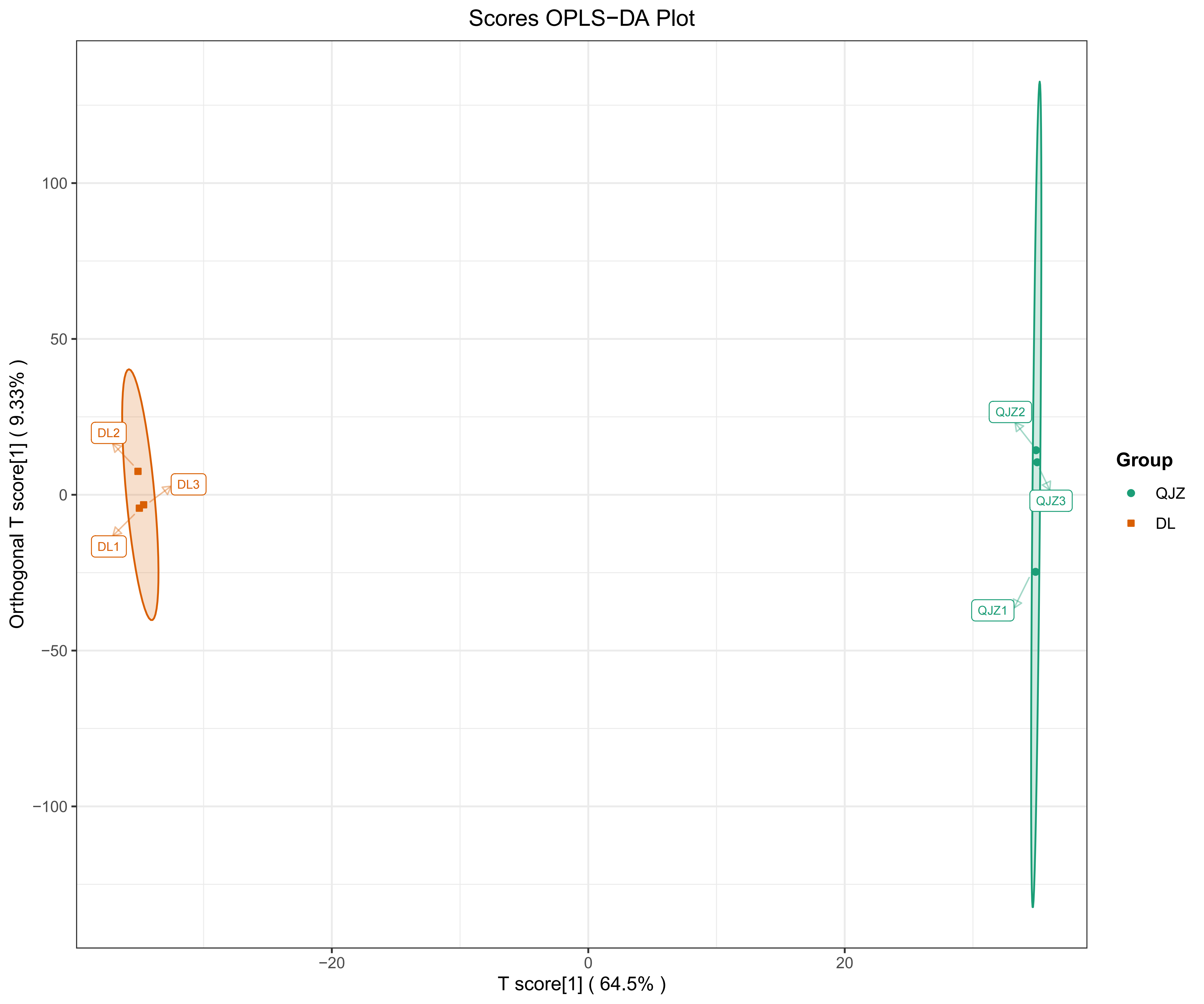

Supplement: Supplementary file 1 [file biomolecules-14-01106-s001.zip › Figure S1 OPLS-DA score plot.png]

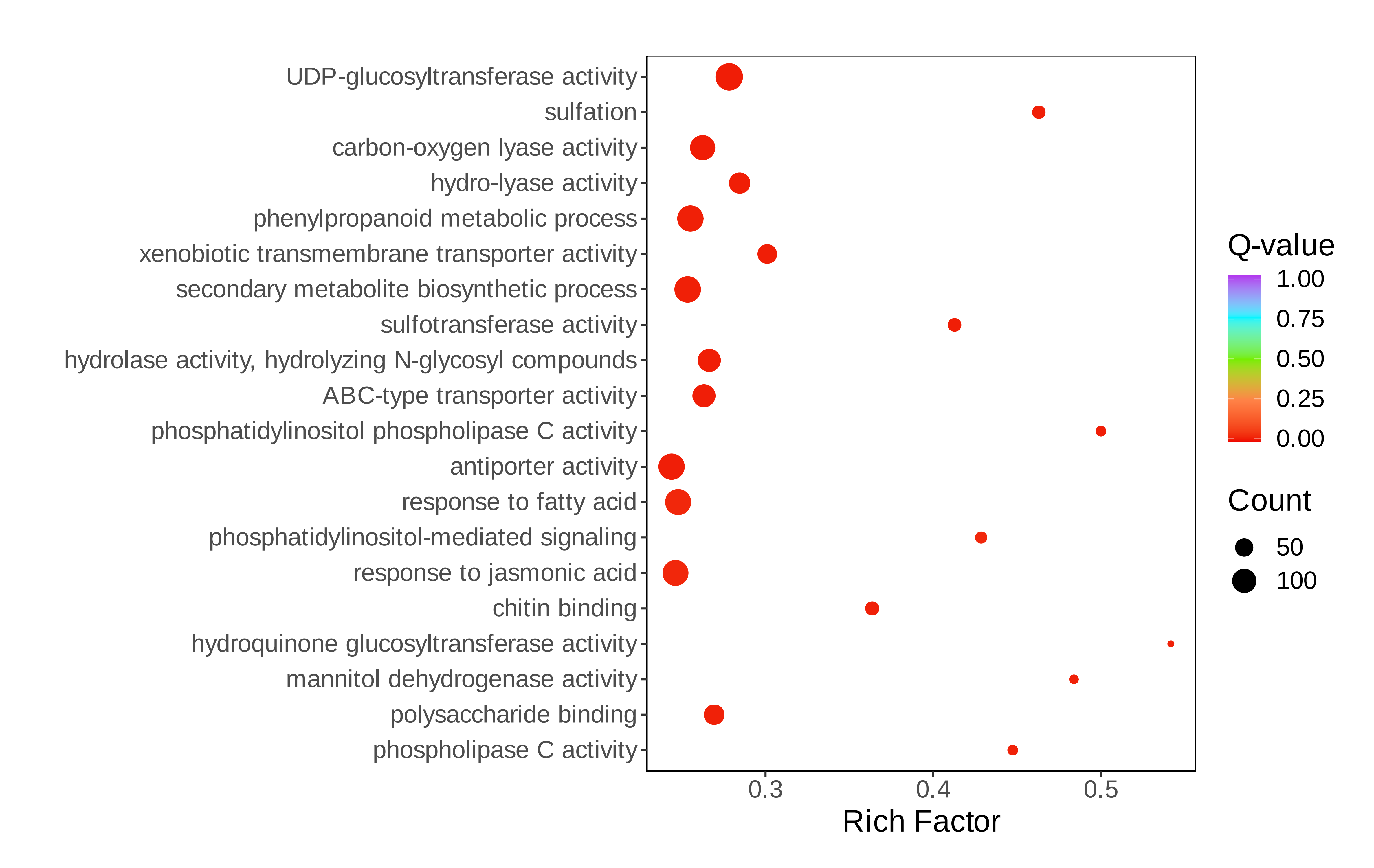

Supplement: Supplementary file 1 [file biomolecules-14-01106-s001.zip › Figure S2 GO enrichment dot plot.png]
